# Supplementary material for: A set of multi-entry identification keys to African frugivorous flies (Diptera, Tephritidae)
Source: Zookeys. 2014 Jul 24;(428):97–108. doi: 10.3897/zookeys.428.7366 (PMC4143993; doi:10.3897/zookeys.428.7366)
Supplement: Supplementary material 4 — Key to Capparimyia [file zookeys-428-097-s004.zip › SF4_ZooKeys_key to Capparimyia/lucid_player/help/help/comparing_entities_the_differences_function.htm]

Comparing entities - The Differences function


# Comparing entities - The Differences function

Suppose you have used the Player to try to identify a specimen, and
at the end of the keying session several entities remain.

One way of trying to decide which of these is correct would be to access
the information (photographs & fact sheets) associated with the entities.
This may allow you to discriminate the entities further.

A better way may be to view a list of features that differ between the
entities. You can access such a list by selecting *Differences*
from the *Entities* menu or by
clicking the Differences  button on the toolbar. These
will open the *Differences* window.

The *Differences* window has
two panels. The upper panel lists all features that differ (that is, are
not scored the same) for the remaining entities. When you click on a specific
feature, the lower panel shows how the entities are scored for the listed
features.

*Differences* in the upper panel
are sorted by their utility for separating the entities, therefore it
is recommended you review the differences from the top down.
